# Association of lung diseases with coronavirus disease 2019 in cancer patients receiving immune checkpoint inhibitors: A multicenter study during national Omicron outbreak in China

**DOI:** 10.1002/ctm2.1497

**Published:** 2023-12-13

**Authors:** Yanlin Li, Tongfei Wang, Yamin Zhang, Miao Li, Xiemin Feng, Rui Xu, Hong Xu, Weihu Xia, Yaning Zhao, Xinli Hou, Hui Wei, Zhiyan Liu, Ying Zan, Bing Zhao, Chunling Liu, Xiaopeng He, Xuan Liang, Mengjie Liu, Lili Jiang, Wenjuan Wang, Yuan Shen, Duolao Wang, Baibing Mi, Yixue Bai, Xin Yu, Xubo Huang, Xiaohui Jia, Xiaolan Fu, Hui Guo, Min Jiao

**Affiliations:** ^1^ Department of Medical Oncology First Affiliated Hospital of Xi'an Jiaotong University Xi'an China; ^2^ Department of Medical Oncology Xi 'an No.3 Hospital the Affiliated Hospital of Northwest University Xi'an China; ^3^ Department of Oncology Xi'an International Medical Center Hospital Xi'an China; ^4^ Department of Medical Oncology Qinghai Provincial Cancer Hospital Fifth People's Hospital of Qinghai Province Xi'an China; ^5^ Department of Medical Oncology Yan'an University Affiliated Hospital Yan'an China; ^6^ Department of Medical Oncology Shaanxi Provincial Cancer Hospital Xi'an China; ^7^ Department of Medical Oncology Baoji Central Hospital Baoji China; ^8^ Department of Medical Oncology Hanzhong Central Hospital Hanzhong China; ^9^ 4th Department of Oncology Affiliated Hospital of Shaanxi University of Chinese Medicine Xianyang China; ^10^ Department of Respiratory and Critical Care Medicine Xi 'an No.3 Hospital the Affiliated Hospital of Northwest University Xi'an China; ^11^ Department of Medical Oncology Second Affiliated Hospital of Xi'an Jiaotong University Xi'an China; ^12^ Department of Pulmonary Medicine Affiliated Cancer Hospital of Xinjiang Medical University Urumqi China; ^13^ Department of Respiratory Medicine Xianyang Central Hospital Xianyang China; ^14^ Department of Epidemiology and Biostatistics School of Public Health Xi'an Jiaotong University Health Science Center Xi'an China; ^15^ Department of Epidemiology and Biostatistics School of Public Health, Xi'an Jiaotong University Health Science Center Xi’an China; ^16^ Key Laboratory of Environment and Genes Related to Diseases Xi'an Jiaotong University Xi'an China; ^17^ Bioinspired Engineering and Biomechanics Center Xi'an Jiaotong University Xi'an China

Dear Editor,

Identifying risk factors of severe manifestation and unfavourable prognosis of coronavirus disease 2019 (COVID‐19) is critical in protecting vulnerable individuals. Gathering patients diagnosed with COVID‐19 from 10 medical centres in China during the national Omicron outbreak, we found that interstitial lung disease (ILD) and atelectasis were associated with a higher risk of worse clinical outcomes, severe symptoms and increased respiratory support in cancer patients receiving immune checkpoint inhibitors (ICIs).

Given that the underlying mechanism of COVID‐19 converges with the pharmacological action of ICIs, there is mounting apprehension about the impact of ICIs on COVID‐19 in cancer patients.^1^, ^2^ What's more, as the main organ affected by severe acute respiratory syndrome coronavirus 2 (SARS‐CoV‐2), the lungs exhibited a robust correlation with COVID‐19 seriousness and outcomes. However, it remained unclear whether pre‐existing lung diseases would affect COVID‐19 outcomes in patients with cancer who have received ICIs. In spite of the epidemic reduction, low‐level Omicron infections may persist worldwide, necessitating increased attention to high‐risk patients with poor prognoses and the provision of detailed health education and better medical resources.^3^

Our study included solid cancer patients with positive SARS‐CoV‐2 tests between December 1, 2022, and February 28, 2023, in 10 medical centres in China^3^ (Table S1). Previous lung diseases, including cancer in the lung (primary and metastatic), ILD, pneumonia, atelectasis and chronic bronchitis/chronic obstructive pulmonary disease (COPD), were evaluated. The primary endpoint was clinical outcomes of COVID‐19, including hospitalization, requiring intensive care and 30‐day mortality (death).^4^, ^5^ COVID‐19 symptoms were also analyzed. All types of respiratory support except nasal cannula were defined as “enhanced respiratory support”. The detail of inclusion and exclusion criteria and statistical method is provided in the Supporting Information.

A total of 626 patients completed the survey, and 212 patients were included after excluding factors such as not receiving ICIs within 6 months before COVID‐19, lack of data and so on (Figure S1). The clinical characteristics are summarized in Table 1 and Table S2. Univariable analysis revealed that age, gender and comorbidities were associated with outcomes and were further analyzed using multivariable models (Table S3).

**TABLE 1:** Baseline characteristics and clinical outcomes of patients included in analysis.

Patients' characteristics (total)	*N* (%)
No. of patients	212 (100.0)
**Demographic characteristic**	
Age, mean, years (range)	60 (25–86)
Sex	
Male	155 (73.1)
Female	57 (26.9)
Smoking status	
Current/former	90 (42.5)
Never	108 (50.9)
Unknown	14 (6.6)
Comorbidities**^†^**	115 (54.2)
**Primary malignancy**	
Lung cancer	110 (51.9)
Esophagogastric cancer	32 (15.1)
Colorectal cancer	17 (8.0)
Others	53 (25.0)
**Anticancer therapy within 6 months before COVID‐19**	
ICIs type	
Anti‐PD‐(L)1	206 (97.2)
Anti‐PD‐(L)1+ anti‐CTLA‐4	6 (2.8)
Combination therapy**^‡,^** ^§^	
None	31 (14.6)
Chemotherapy	145 (68.4)
Targeted therapy	56 (26.4)
ICI treatment duration, median (IQR), days	139.5 (45.8–422.5)
Last ICI before COVID‐19, median (IQR), days	28 (14‐76)
Radiotherapy in lungs	11 (5.2)
**Previous lung disease before COVID‐19**	
Cancer in lung	139 (65.6)
Primary lung cancer	110 (51.9)
Metastatic lung cancer	29 (13.7)
ILD	77 (36.3)
Idiopathic interstitial pneumonia	59 (27.8)
ICI‐pneumonitis	12 (5.7)
Radiation pneumonitis	6 (2.8)
Pneumonia (infective)	40 (18.9)
Atelectasis	38 (17.9)
Non‐obstructive atelectasis	24 (11.3)
Obstructive atelectasis	14 (6.6)
Chronic bronchitis/COPD	17 (8.0)
Chronic bronchitis	8 (3.8)
COPD	9 (4.2)

^†^ Excluding pulmonary comorbidities.

^‡^ Percentages could sum to > 100% because categories are not mutually exclusive.

^§^ Systematic anti‐cancer therapy.

Abbreviations: ICI, immune‐checkpoint inhibitor; COPD, chronic obstructive pulmonary disease; CTLA‐4, cytotoxic T‐lymphocyte‐associated protein 4; ILD, interstitial lung disease; IQR, interquartile range; PD‐(L)1, programmed death (ligand)−1.

The percentage of pre‐existing lung diseases and their coexistence is shown in Figure S2. Multivariable analysis revealed that ILD was associated with a higher risk of hospitalization (adjusted odds ratio [aOR] 2.06 [1.10, 3.87], *p* = .024) and intensive care (aOR 2.79 [1.15, 6.78], *p* = .023). Moreover, among patients with ILD, idiopathic interstitial pneumonia was related to an increased risk of hospitalization (aOR 2.10 [1.09, 4.05], *p* = .028) and death (aOR 4.16 [1.23, 13.99], *p* = .021). Atelectasis was related to an elevated risk of hospitalization (aOR 2.27 [1.07, 4.83], *p* = .034). However, cancer in the lung, pneumonia and chronic bronchitis/COPD were not found to be associated with COVID‐19 clinical outcomes (Figure 1).

**FIGURE 1:**
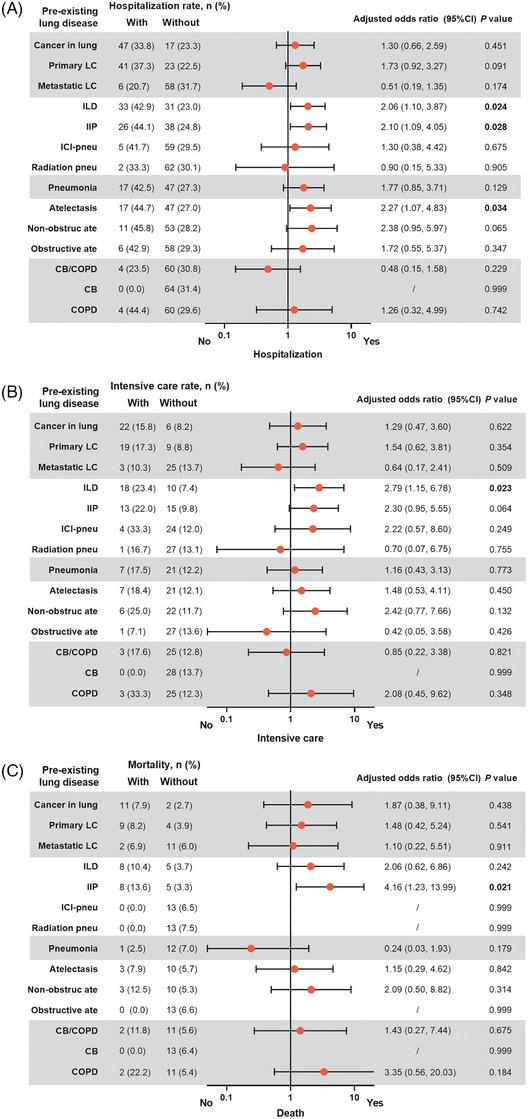
**Association of pre‐existing lung diseases with clinical outcomes of coronavirus disease 2019 (COVID‐19)**. Adjusted odds ratio (ORs) for the association of pre‐existing lung diseases with hospitalization (A), intensive care (B), and death (C) related to COVID‐19. Adjusting covariates for each clinical outcome were age, sex and comorbidities at COVID‐19 diagnosis. ORs were calculated using multivariate logistic regression. Error bars represent 95% CIs. *CI, confidence interval; LC, lung cancer; ILD, interstitial lung disease; IIP, idiopathic interstitial pneumonia; ICI, immune checkpoint inhibitor; pneu, pneumonitis; non‐obstruc ate, non‐obstructive atelectasis; obstructive ate, obstructive atelectasis; CB, chronic bronchitis; COPD, chronic obstructive pulmonary disease*.

The symptoms are presented in Table S4. Patients with cancer in the lung, ILD or chronic bronchitis/COPD had higher incidences of fever (cancer in the lung, *p* = .019; ILD, *p* = .026; chronic bronchitis/COPD, *p* = .031). Patients with ILD or atelectasis had a higher rate of dyspnea (ILD, *p* = .003; atelectasis, *p* < .001) (Figure 2). Subgroup analysis illustrated that patients with idiopathic interstitial pneumonia had a higher rate of fever (*p* = .013), and patients with idiopathic interstitial pneumonia or non‐obstructive atelectasis had a higher incidence of dyspnea (idiopathic interstitial pneumonia, *p* = .040; non‐obstructive atelectasis, *p* < .001) (Figure S3). Additionally, 45 (21.2%) patients required respiratory support (Table S5). The correlation between pre‐existing lung diseases and respiratory support is shown in Figure 3A,B and Figure S4A,B. The results indicated that patients with ILD or atelectasis had a higher rate of respiratory support (ILD, *p* < .001; atelectasis, *p* = .031). Idiopathic interstitial pneumonia, one of the ILDs, was associated with a higher rate of respiratory support (*p* = .005). There was no difference in the requirement for enhanced respiratory support between patients with and without lung diseases. We also analyzed the oxygenation index in patients receiving any kind of respiratory support and found that patients with COPD had a lower oxygenation index compared to patients without COPD (*p* = .049) (Figure 3C and Figure S4C).

**FIGURE 2:**
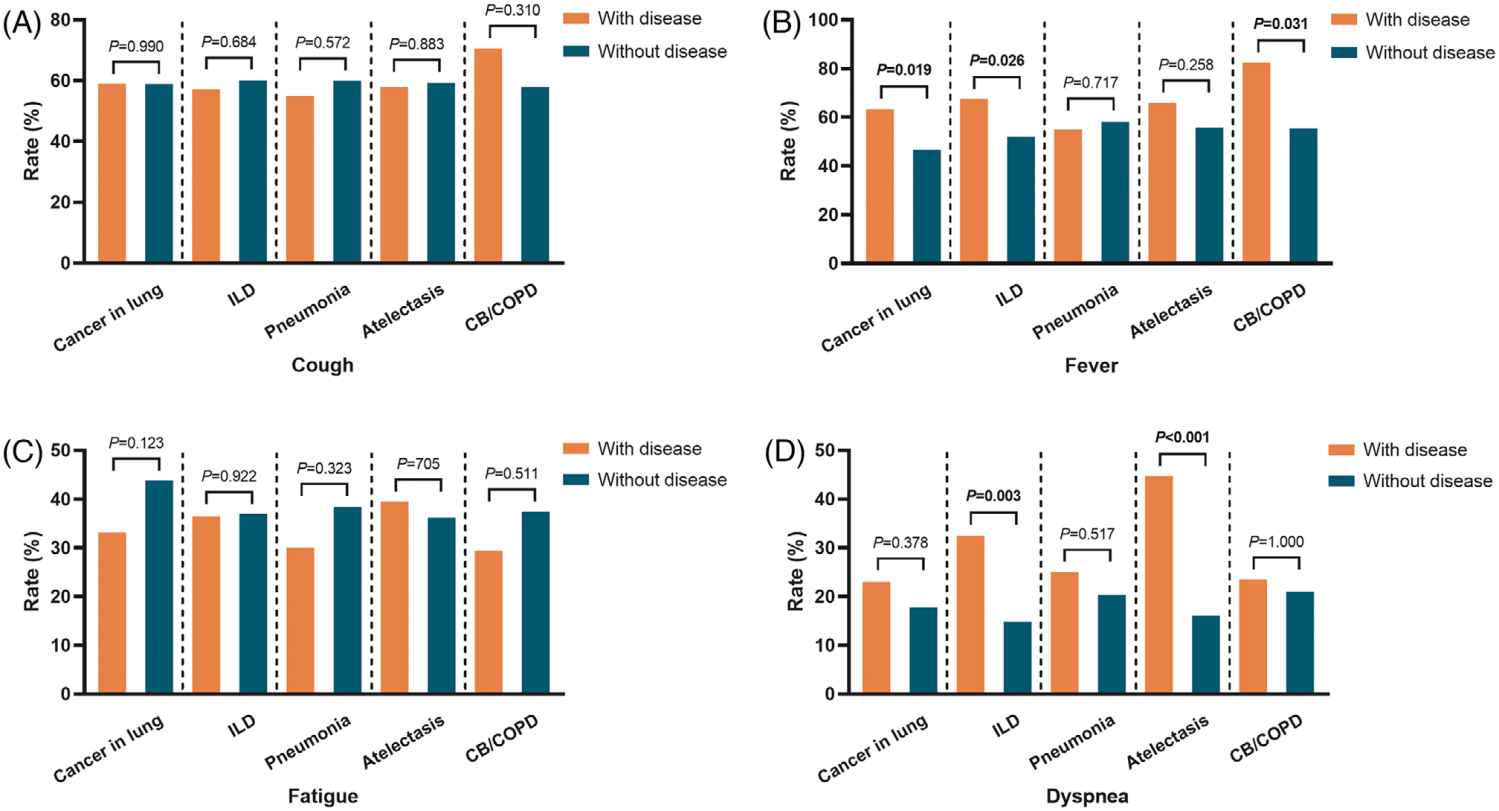
**Association of pre‐existing lung diseases with coronavirus disease 2019 (COVID‐19) symptoms**. Rate of cough (A), fever (B), fatigue (C) and dyspnea (D) between patients with and without pre‐existing lung disease. *p‐*Values were calculated using the chi‐square test. *ILD, interstitial lung disease; CB, chronic bronchitis; COPD, chronic obstructive pulmonary disease*.

**FIGURE 3:**
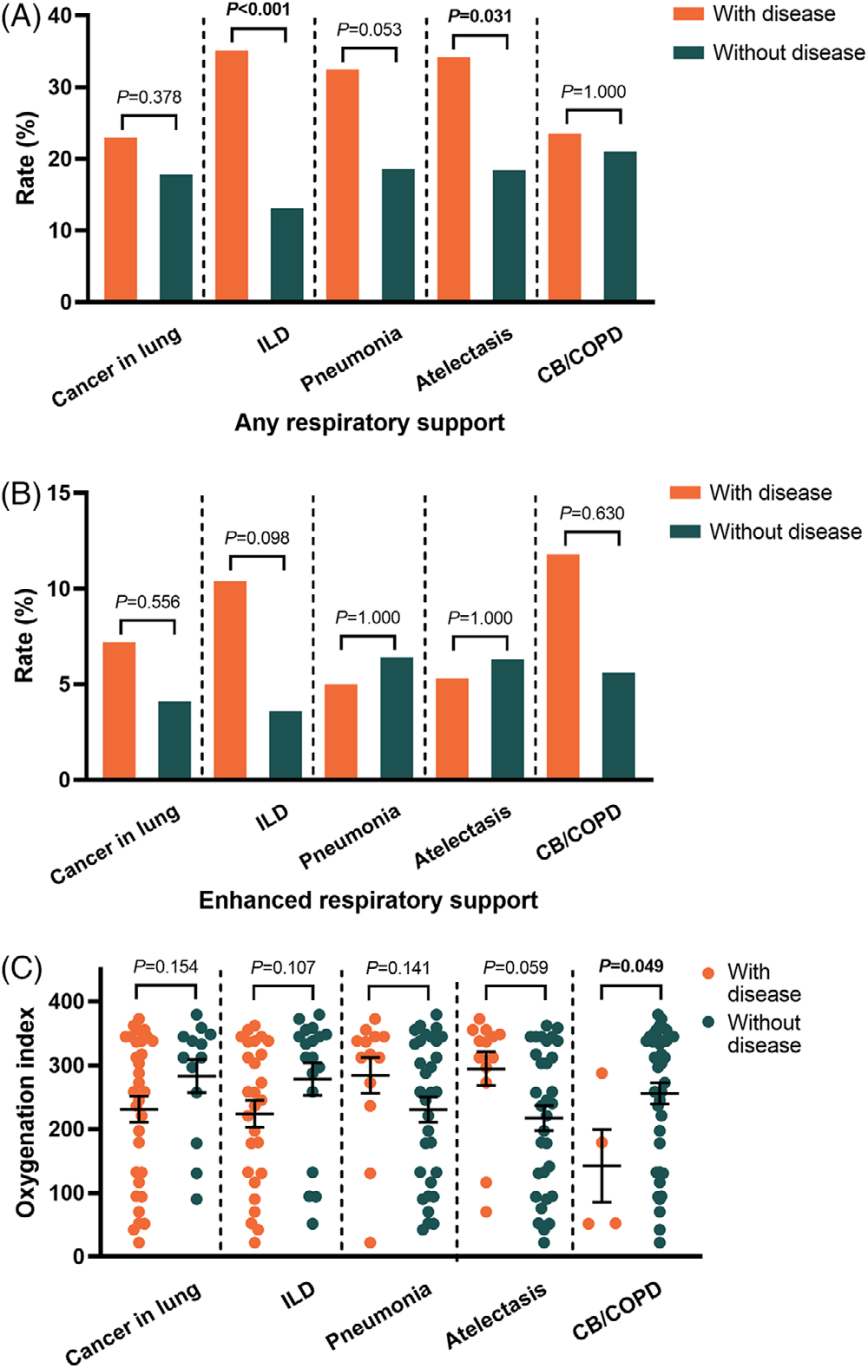
**Association of pre‐existing lung diseases with the requirement of respiratory support due to coronavirus disease 2019 (COVID‐19)**. Rate of any respiratory support (A) and enhanced respiratory support (B) between patients with and without pre‐existing lung disease. Oxygenation index between patients with and without pre‐existing lung disease in patients receiving any respiratory support (C). *p‐*Values were calculated using the chi‐square test (A, B) and unpaired t‐test (C). Bars indicate the mean and SEM (C). *ILD, interstitial lung disease; CB, chronic bronchitis; COPD, chronic obstructive pulmonary disease; SEM, standard error of the mean*.

Our study provides a contemporary understanding of the impact of pre‐existing lung diseases on COVID‐19. To our knowledge, this is the first analysis focusing on patients treated with ICIs and multiple pre‐existing lung diseases in China during the Omicron pandemic. These findings can help better protect vulnerable individuals in future COVID‐19 and other virus epidemic.

Although some biomarkers have been found to be valuable in forecasting the seriousness and prognosis of COVID‐19, the association between patients` pre‐existing comorbidities and COVID‐19 is also noteworthy.^6^ Consistent with the population in the community, ILD was notably correlated with a raised risk of severe outcomes in patients receiving ICIs.^7^ ILD may serve as a marker of pulmonary inflammatory activity, which can be further interfered with by ICIs, leading to disturbances in the antiviral immune response.^8^ Patients with ILD had a higher probability of experiencing fever and dyspnea, potentially due to stronger antiviral immune response and more severe damage to the alveoli and bronchial tubes. The pulmonary inflammation and immune impairment caused by ILD may be more severe in patients treated with ICIs and more susceptible to SARS‐CoV‐2 viruses. As the result showed, patients with atelectasis had worse outcomes of COVID‐19, which may be attributed to causes of atelectasis, such as malignant pleural effusion and bronchial obstruction, leading to further lung deterioration.^9^ Atelectasis can also induce local tissue inflammation and immune response disorders, increasing the vulnerability to infection.^10^

In conclusion, we found that ILD and atelectasis are correlated with an elevated likelihood of adverse outcomes in cancer patients receiving ICIs. These findings amplify the importance of maintaining an urgent focus on patients with pre‐existing lung diseases and enhancing the prevention and treatment approaches in the prevalence of SARS‐CoV‐2 and other respiratory viruses. Further research is needed to better understand the mechanisms.

## AUTHOR CONTRIBUTIONS

Conception and design of the work: Yanlin Li and Tongfei Wang Data collection/management: Yanlin Li, Tongfei Wang, Yamin Zhang, Miao Li, Xiemin Feng, Rui Xu, Hong Xu, Weihu Xia, Yaning Zhao, Xinli Hou, Hui Wei, Zhiyan Liu, Ying Zan, Bing Zhao, Chunling Liu, Xiaopeng He, Xuan Liang, Yixue Bai, Xin Yu and Xubo Huang Statistical analysis: Yuan Shen and Baibing Mi Data interpretation: Mengjie Liu, Lili Jiang, Wenjuan Wang and Xiaohui Jia Drafting the article: Yanlin Li and Hui Guo Critical revision of the article: Duolao Wang, Hui Guo and Min Jiao Final approval of the version: Hui Guo and Min Jiao

## CONFLICT OF INTEREST STATEMENT

The authors declare no conflict of interest.

## FUNDING INFORMATION

This work was supported by CSCO‐MSD Innovation Fund (Y‐MSD2020‐0247); Guangdong Association of Clinical Trials (GACT)/Chinese Thoracic Oncology Group (CTONG) and Guangdong Provincial Key Lab of Translational Medicine in Lung Cancer (No.2017B0303141020); Fundamental Research Funds for Central Universities, Interdisciplinary Team of Tumor Immunotherapy and Physical Microenvironment (xtr062021001) and The Clinical Research Award of the First Affiliated Hospital of Xi'an Jiaotong University (No.XJTU1AF‐CRF‐2022‐020). The funding sources were involved in the writing of the report.

### ETHICS STATEMENT

This study was approved by the Ethics Committee of First Affiliated Hospital of Xi'an Jiaotong University (XJTU1AF2023LSK‐157). All patients signed a written informed consent.

## Supporting information

Supporting Information

Supporting Information

Supporting Information

Supporting Information

Supporting Information

Supporting Information

## Data Availability

The raw data supporting the conclusions of this article will be made available by the authors, without undue reservation.
