# Supplementary figures and images for: Association of lung diseases with coronavirus disease 2019 in cancer patients receiving immune checkpoint inhibitors: A multicenter study during national Omicron outbreak in China
Source: Clin Transl Med. 2023 Dec 13;13(12):e1497. doi: 10.1002/ctm2.1497 (PMC10719537; doi:10.1002/ctm2.1497)

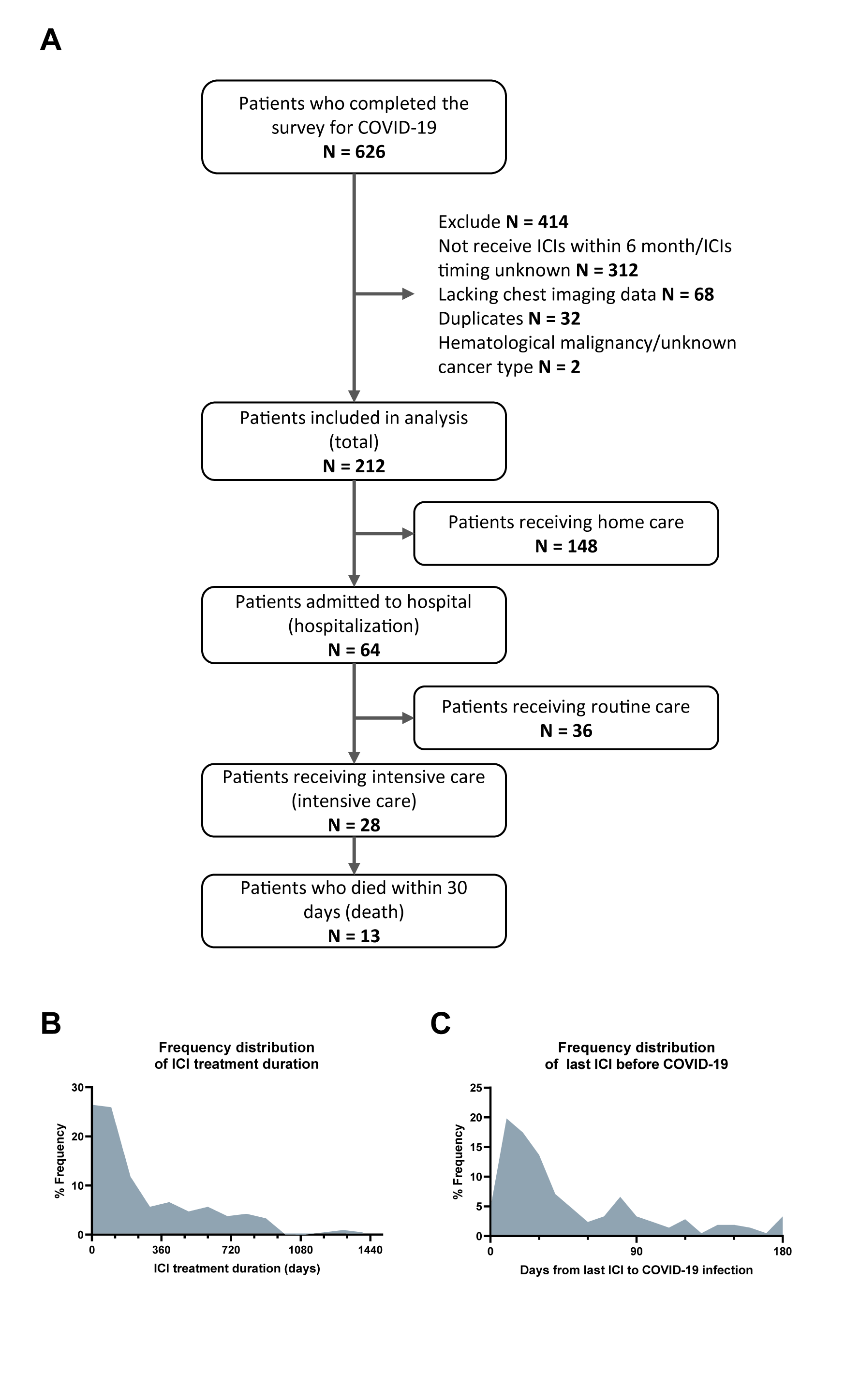

Supplement: Supplementary file 1 — Supporting Information [file CTM2-13-e1497-s001.tif]

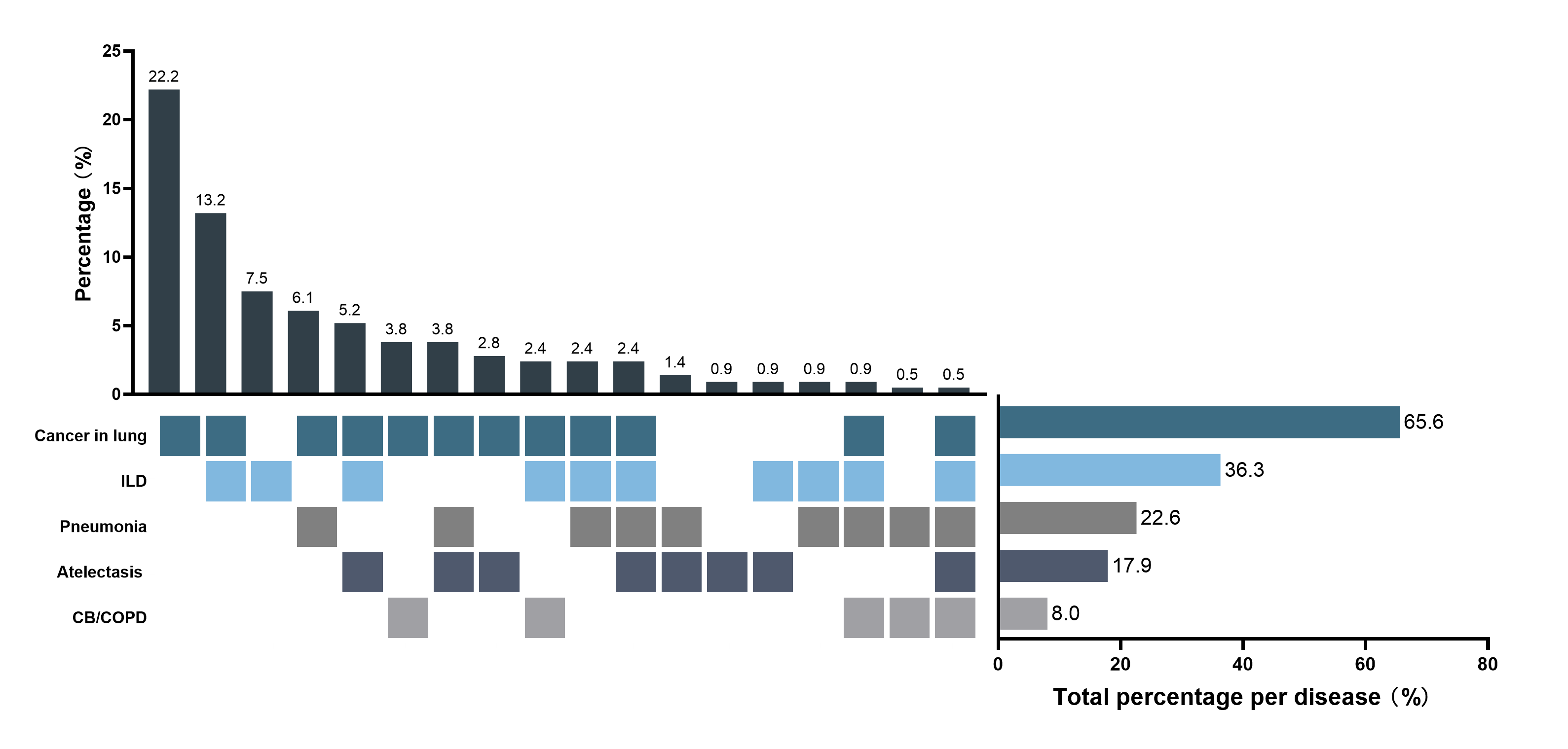

Supplement: Supplementary file 2 — Supporting Information [file CTM2-13-e1497-s004.tif]

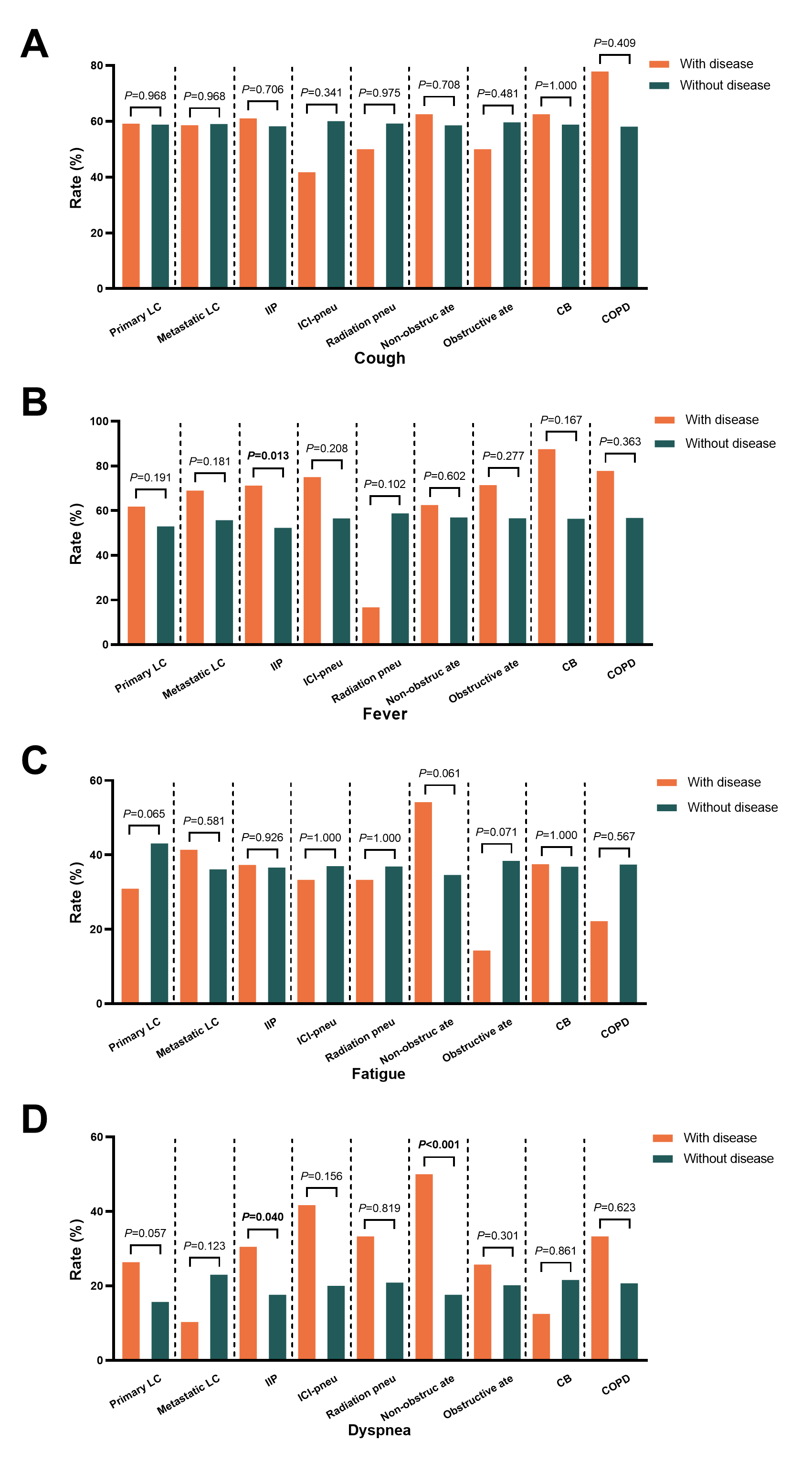

Supplement: Supplementary file 3 — Supporting Information [file CTM2-13-e1497-s003.tif]

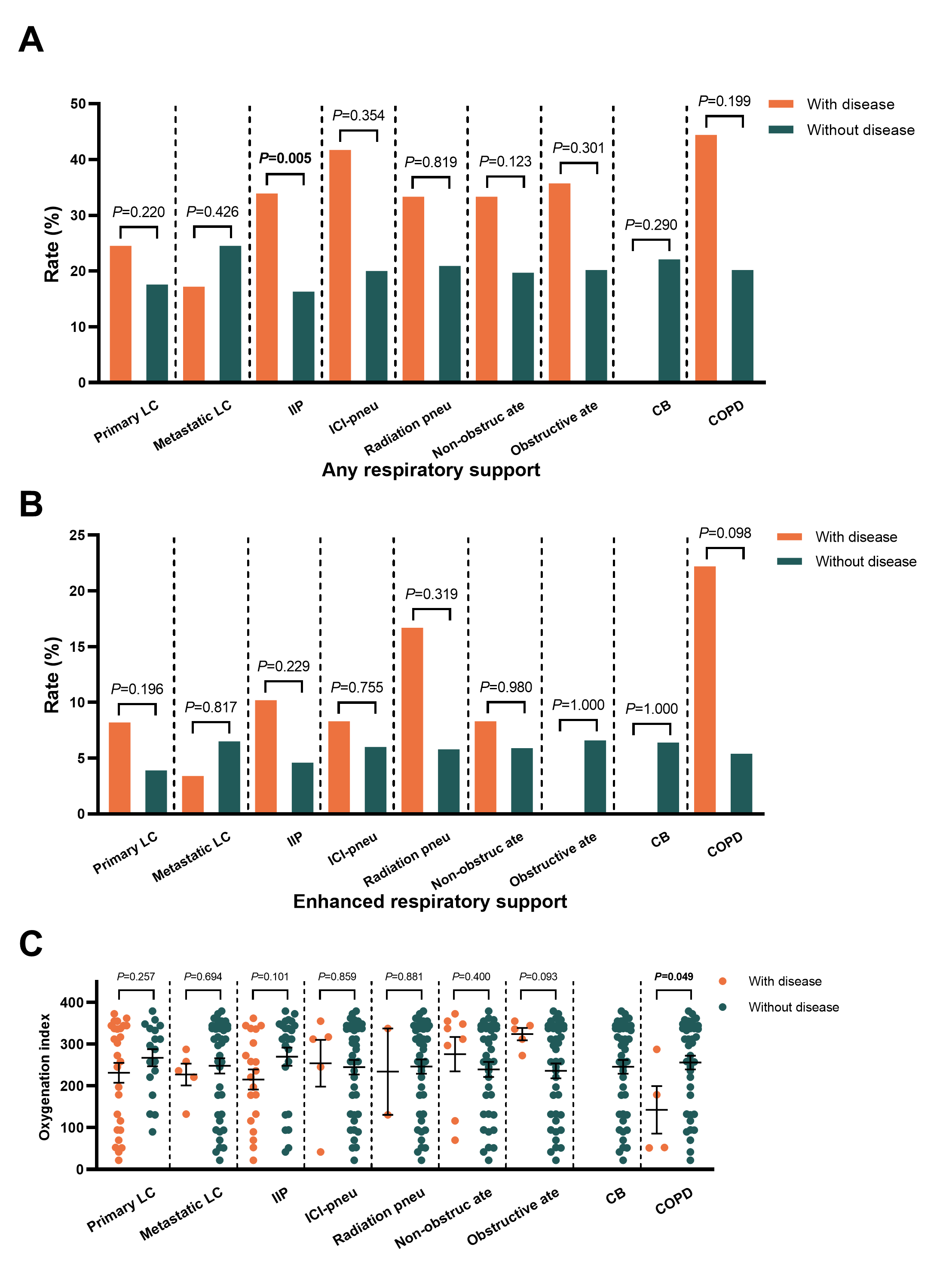

Supplement: Supplementary file 4 — Supporting Information [file CTM2-13-e1497-s002.tif]
