# Supplementary material for: Association of lung diseases with coronavirus disease 2019 in cancer patients receiving immune checkpoint inhibitors: A multicenter study during national Omicron outbreak in China
Source: Clin Transl Med. 2023 Dec 13;13(12):e1497. doi: 10.1002/ctm2.1497 (PMC10719537; doi:10.1002/ctm2.1497)
Supplement: Supplementary file 5 — Supporting Information [file CTM2-13-e1497-s006.docx]

**Legends of online figures**

**Figure E1. Study flow diagram showing patients enrollment and severity of COVID-19**. *ICI, immune checkpoint inhibitor.*

**Figure E2. UpSet plot of individual previous lung diseases and their coexistence.** Each row represents the percentage of one pre-existing lung disease before COVID-19 infection and each column represents the percentage of coexistence of one or more lung diseases. A column with a colorful square represents a lung disease and columns with multiple colorful squares represent coexistence of lung diseases. *ILD, interstitial lung disease; CB, chronic bronchitis; COPD, chronic obstructive pulmonary disease.*

**Figure E3.** **Association of pre-existing lung diseases (subgroups) with COVID-19 symptoms.** Rate of cough (A), fever (B), fatigue (C) and dyspnea (D) between patients with and without pre-existing lung disease. *P* values were calculated using chi-square test or Fisher’s exact test. *LC, lung cancer; IIP, idiopathic interstitial pneumonia; ICI, immune checkpoint inhibitor; pneu, pneumonitis; non-obstruc ate, non-obstructive atelectasis; obstructive ate, obstructive atelectasis; CB, chronic bronchitis; COPD, chronic obstructive pulmonary disease.*

**Figure E4. Association of pre-existing lung diseases (subgroups) with the requirement of respiratory support due to COVID-19.** Rate of any respiratory support (A) and enhanced respiratory support (B) between patients with and without pre-existing lung disease. Oxygenation index between patients with and without pre-existing lung disease in patients receiving any respiratory support (C). *P* values were calculated using chi-square test or Fisher’s exact test (A, B) and unpaired t test (C). Bars indicate the mean and SEM (C). *LC, lung cancer; IIP, idiopathic interstitial pneumonia; ICI, immune checkpoint inhibitor; pneu, pneumonitis; non-obstruc ate, non-obstructive atelectasis; obstructive ate, obstructive atelectasis; CB, chronic bronchitis; COPD, chronic obstructive pulmonary disease.*
