# Supplementary material for: Association of lung diseases with coronavirus disease 2019 in cancer patients receiving immune checkpoint inhibitors: A multicenter study during national Omicron outbreak in China
Source: Clin Transl Med. 2023 Dec 13;13(12):e1497. doi: 10.1002/ctm2.1497 (PMC10719537; doi:10.1002/ctm2.1497)
Supplement: Supplementary file 6 — Supporting Information [file CTM2-13-e1497-s005.docx]

| **Table E1. List of participating centers and eligible patients** | | | |
| --- | --- | --- | --- |
| Participating clinical center | City | No. of patients | (%) |
| The First Affiliated Hospital of Xi'an Jiaotong University | Xi’an | 110 | 51.9 |
| Xi’an International Medical Center Hospital | Xi’an | 34 | 16.0 |
| The Third Hospital of Xi’an | Xi’an | 23 | 10.8 |
| The Second Affiliated Hospital of Xi’an Jiaotong University | Xi’an | 14 | 6.6 |
| The Fifth People’s Hospital of Qinghai | Xining | 11 | 5.2 |
| Yan’an University Affiliated Hospital | Yan’an | 7 | 3.3 |
| Shaanxi Provincial Cancer Hospital | Xi’an | 6 | 2.8 |
| Baoji Central Hospital | Baoji | 3 | 1.4 |
| Hanzhong Central Hospital | Hanzhong | 2 | 0.9 |
| The Affiliated Hospital of Shaanxi University of Chinese Medicine | Xianyang | 2 | 0.9 |

| **Table E2. Details of comorbidities** | |
| --- | --- |
| Category of comorbidity | No. of patient (%) |
| Cardiovascular | 56 (26.4) |
| Endocrine | 36 (17.0) |
| Liver | 18 (8.5) |
| Renal | 5 (2.4) |
| Hematological | 3 (1.4) |
| Nervous | 3 (1.4) |
| Any**^†^** | 115 (54.2) |
| † Any is less than the sum of the above items because some patients had more than one comorbidity. | |

| **Table E3. Univariable logistic analysis of demographic characteristic and anticancer therapy** | | | | | | |
| --- | --- | --- | --- | --- | --- | --- |
|  | **Hospitalization** | | **Intensive care** | | **Death** | |
|  | **OR** | ***P* value** | **OR** | ***P* value** | **OR** | ***P* value** |
| Age, < 60 vs. ≥ 60 | 0.62 (0.34, 1.15)) | 0.132 | 0.21 (0.07, 0.62) | **0.005** | 0.11 (0.01, 0.88) | **0.037** |
| Sex, male vs. female | 2.93 (1.34, 6.43) | **0.007** | 5.54 (1.27, 24.17) | **0.023** | 4.70 (0.60, 37.00) | 0.142 |
| Smoking status,  current/former vs never | 1.82 (0.99, 3.33) | 0.052 | 1.24 (0.56, 2.75) | 0.603 | 1.43 (0.46, 4.43) | 0.531 |
| Comorbidities, yes vs. no | 1.96 (1.07, 3.60) | **0.030** | 6.13 (2.05, 18.37) | **0.001** | 5.02 (1.09, 23.25) | **0.039** |
| ICIs type,  anti-PD- (L)1 vs. anti-PD- (L)1+ anti-CTLA-4 | 0.42 (0.08, 2.14) | 0.297 | 0.75 (0.09, 6.70) | 0.800 | / | 0.999 |
| Combination therapy |  |  |  |  |  |  |
| None, yes vs. no | 1.57 (0.71, 3.46) | 0.266 | 1.32 (0.46, 3.78) | 0.604 | 0.47 (0.06, 3.75) | 0.475 |
| Chemotherapy, yes vs. no | 0.62 (0.33, 1.15) | 0.126 | 0.57 (0.25, 1.28) | 0.173 | 0.72 (0.23, 2.30) | 0.724 |
| Targeted, yes vs. no | 0.90 (0.46, 1.76) | 0.759 | 0.90 (0.46, 1.76) | 0.759 | 1.26 (0.37, 4.25) | 0.714 |
| Last ICI before COVID-19,  < 90 days vs. 90-180 days | 1.43 (0.63, 3.23) | 0.394 | 1.31 (0.43, 4.03) | 0.636 | 1.17 (0.25, 5.53) | 0.839 |
| ICI treatment duration,  < 360 days vs. ≥ 360 days | 1.04 (0.55, 1.96) | 0.917 | 2.17 (0.79, 5.99) | 0.135 | 2.49 (0.54, 11.57) | 0.245 |
| Radiotherapy in lungs,  yes vs. no | 2.96 (0.87, 2.96) | 0.083 | 0.644 (0.079, 5.24) | 0.681 | / | 0.999 |
| *ICI, immune checkpoint inhibitor; PS, performance status; PD-(L)1, programmed death (ligand )-1; CTLA-4,* *cytotoxic T-lymphocyte-associated protein 4;* | | | | | | |

| **Table E4. Details of COVID-19 symptoms** | |
| --- | --- |
| Symptom of COVID-19 | No. of patient (%)**^†^** |
| Cough | 125 (59.0) |
| Fever | 122 (57.5) |
| Fatigue | 78 (36.8) |
| Dyspnea | 45 (21.2) |
| Sore throat | 37 (17.5) |
| Gastrointestinal symptom | 25 (11.8) |
| Headache | 21 (9.9) |
| Others | 6 (28.3) |
| † Percentages could sum to > 100% because categories are not mutually exclusive. | |

| **Table E5. Details of respiratory support** | |
| --- | --- |
| Categories of respiratory support | No. of patient (%) |
| No respiratory support | 167 (78.8) |
| Nasal cannula | 32 (15.1) |
| High-flow nasal cannula | 7 (3.3) |
| Non-rebreather mask | 1 (0.5) |
| Non-invasive positive-pressure ventilation | 2 (0.9) |
| Intubation with mechanical ventilation | 3 (1.4) |
